# Supplementary material for: Assessing the Impact of SARS-CoV-2 Lineages and Mutations on Patient Survival
Source: Viruses. 2022 Aug 27;14(9):1893. doi: 10.3390/v14091893 (PMC9500738; doi:10.3390/v14091893)
Supplement: Supplementary file 1 [file viruses-14-01893-s001.zip › viruses-1876748-supplementary.pdf]

Assessing the impact SARS-CoV-2 lineages and mutations on patient survival.

Supplementary Material

Content

Supplementary Figure S1. 2

Supplementary Figure S2. 3

Supplementary Figure S3. 4

Supplementary Figure S4. 5

Supplementary Figure S5. 7

Supplementary Table S1. 8

Supplementary Table S2. 25

The Andalusian COVID-19 sequencing initiative 28

Supplementary Figure S1.

Circulation of the five SARS-CoV-2 variants eligible for the causal analysis in A) Andalusia, B) Spain, C) France, D) United Kingdom, and E) Portugal.

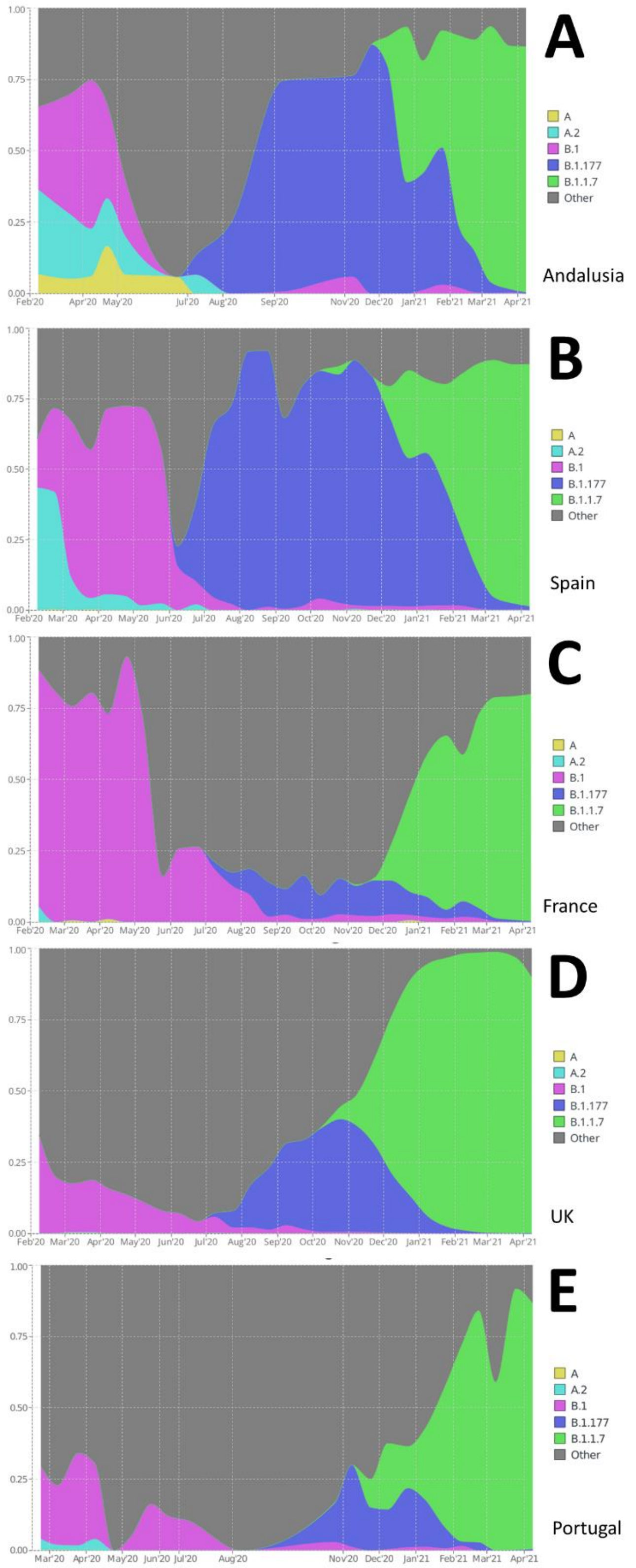

Supplementary Figure S2.

Log Hazard ratios estimated for the 69 nucleotide mutations eligible for the causal analysis using the two approaches described in the text (the closed form estimator and the bootstrap). For each analysis an estimate of the LHR along with a 95% confidence interval and a p-value (FDR adjusted) of significance is provided.

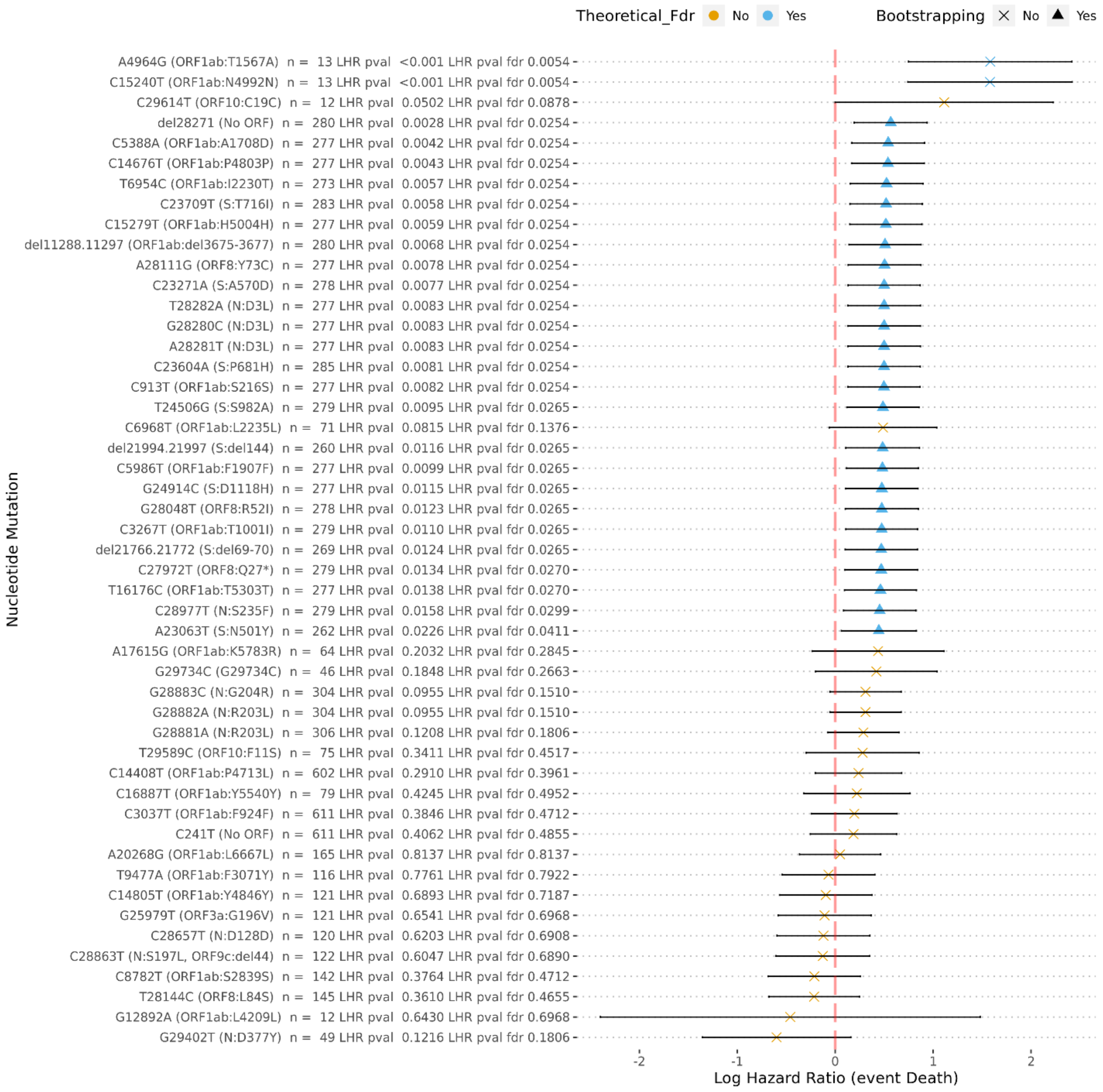

Supplementary Figure S3.

Correlations among the mutations in the SARS-CoV-2 genome significantly associated to patient survival.

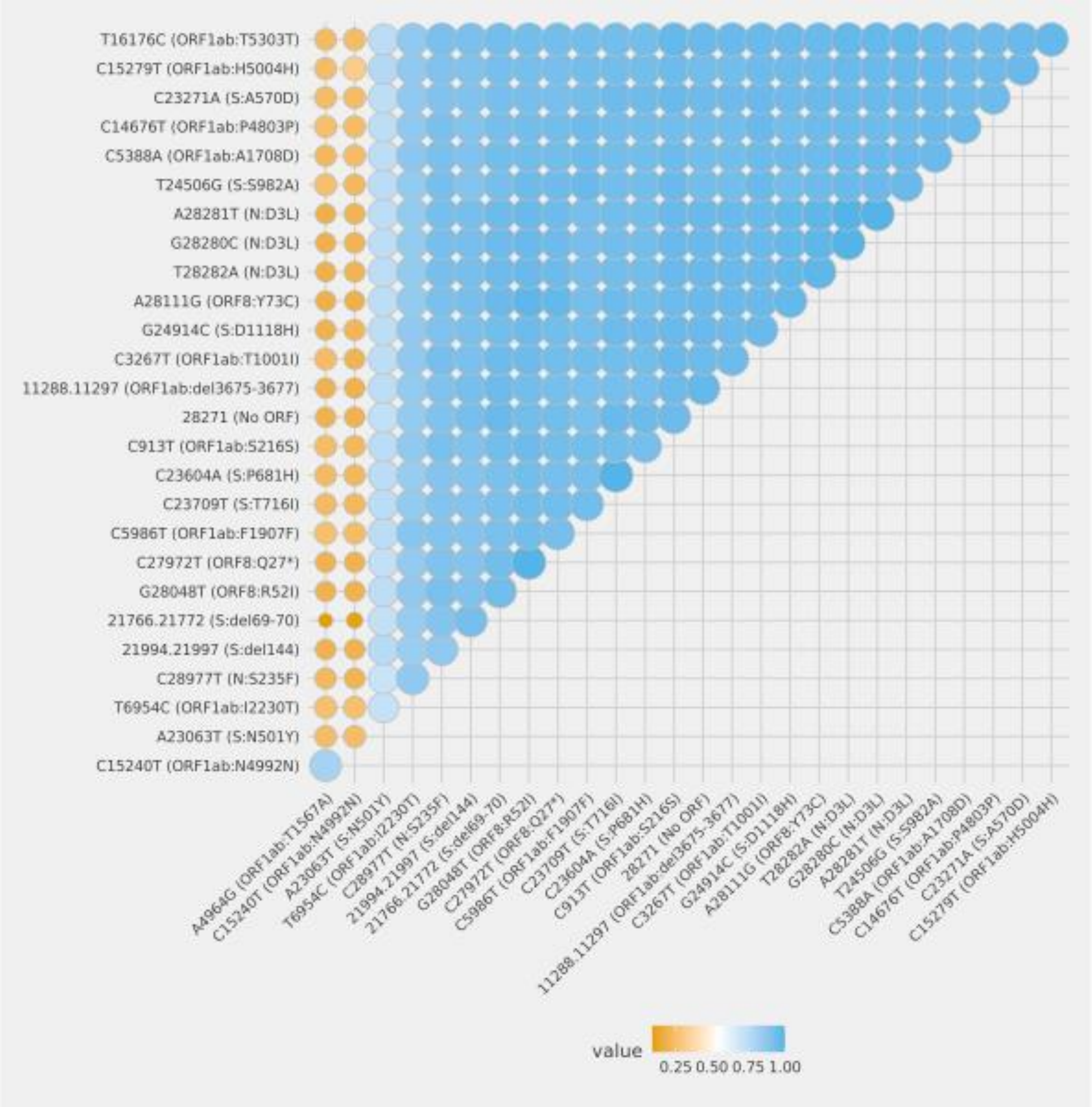

Supplementary Figure S4.

Mutations occurring during the period studied (from 19th February 2020 to 30th April 2021) represented over the variants in which they appear in two phylogenetic formats. First column contains the mutation. Second column accounts for the evolutionary rates. Third column with the time at which every variant was sampled from a patient.

Mutation	Clock-like phylogeny	Phylogeny with time in the X axis
ORF1ab: A1708D	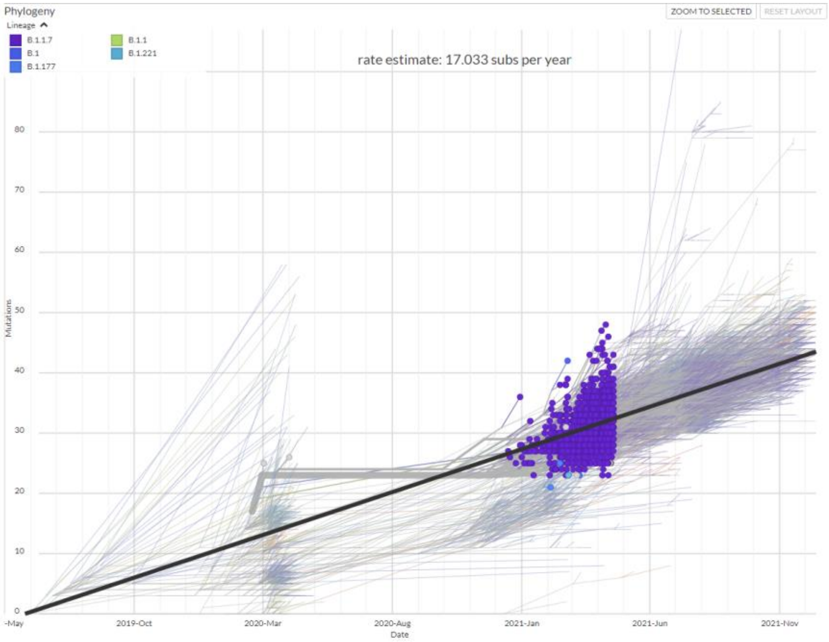 A	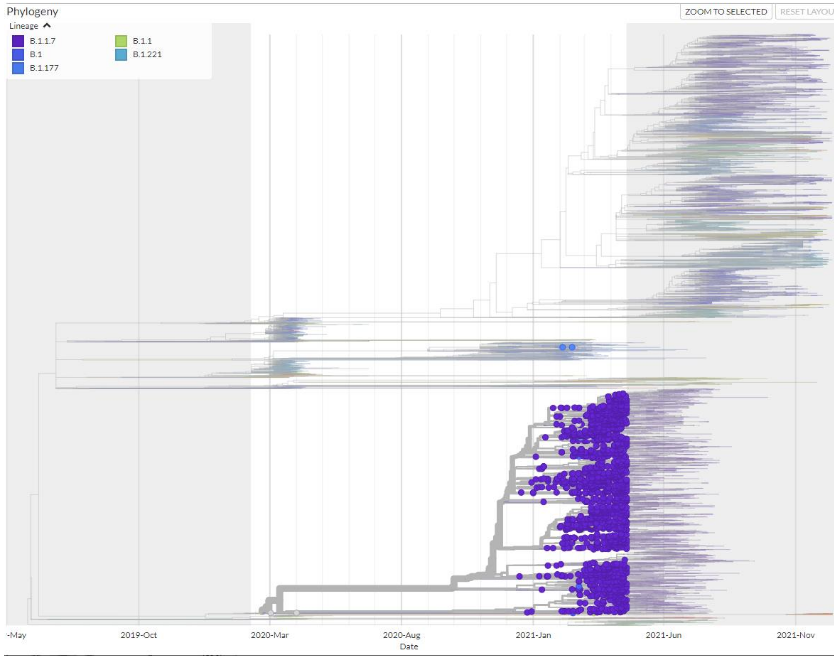 B

ORF1ab:T1567A

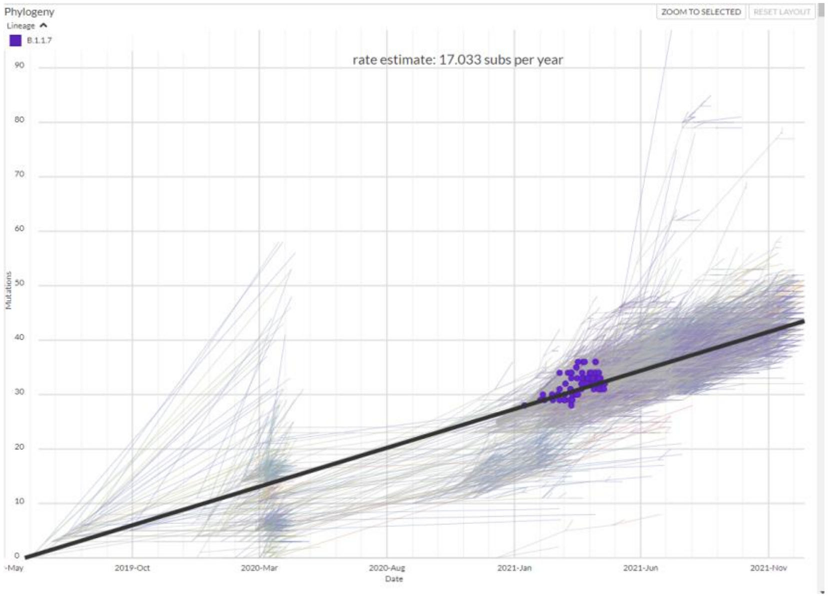

C

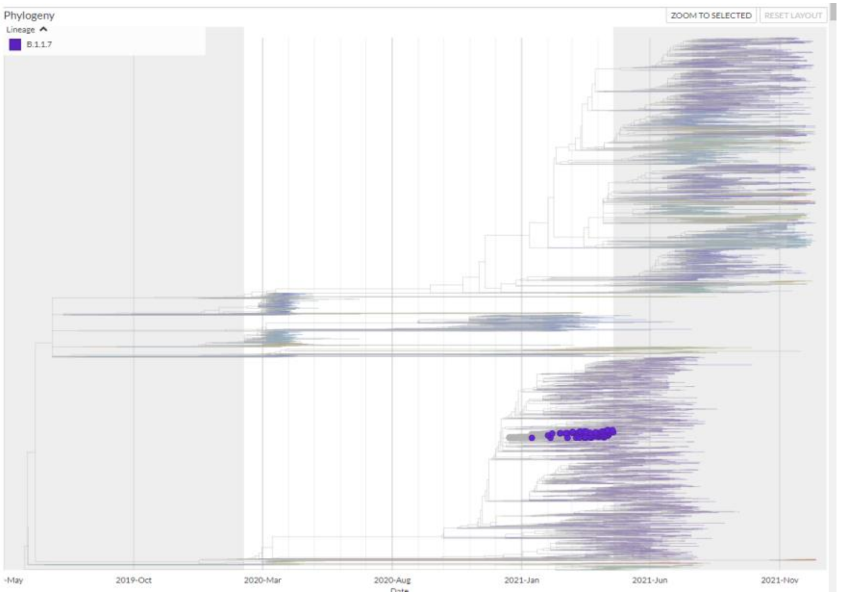

D

N:D377Y

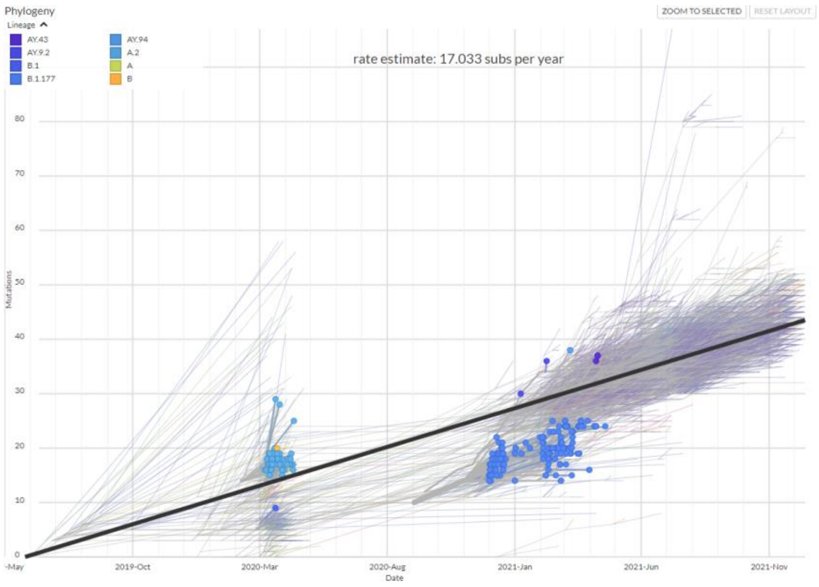

E

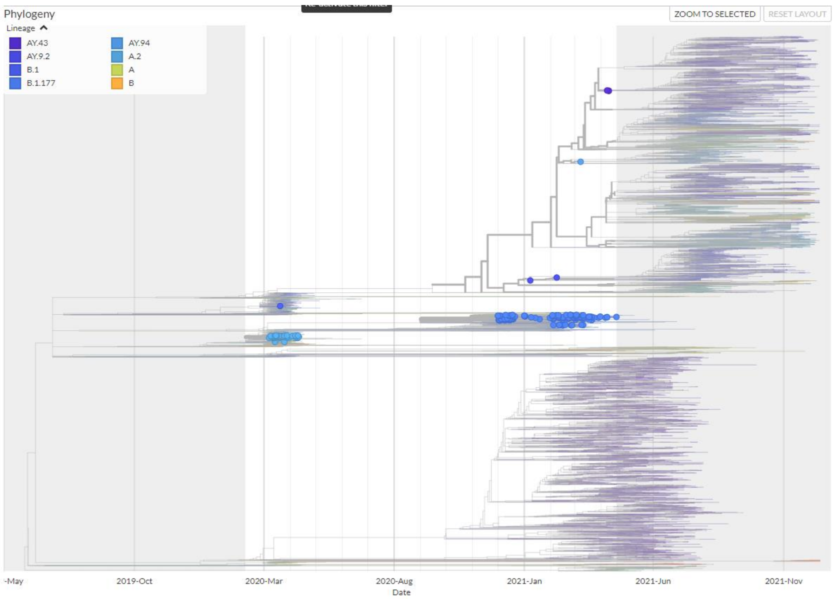

F

Supplementary Figure S5.

Presence of the mutation N:D377Y in the different SARS-CoV-2 viral genomes sampled in Andalusia according to the Genomic surveillance circuit of Andalusia. The upper branch corresponds to the delta variant and subtypes and the lower one to the almost extinct alpha variant.

See http://nextstrain.clinbioinfosspa.es/SARS-COV-2-all?branchLabel=none&gt=N.377Y

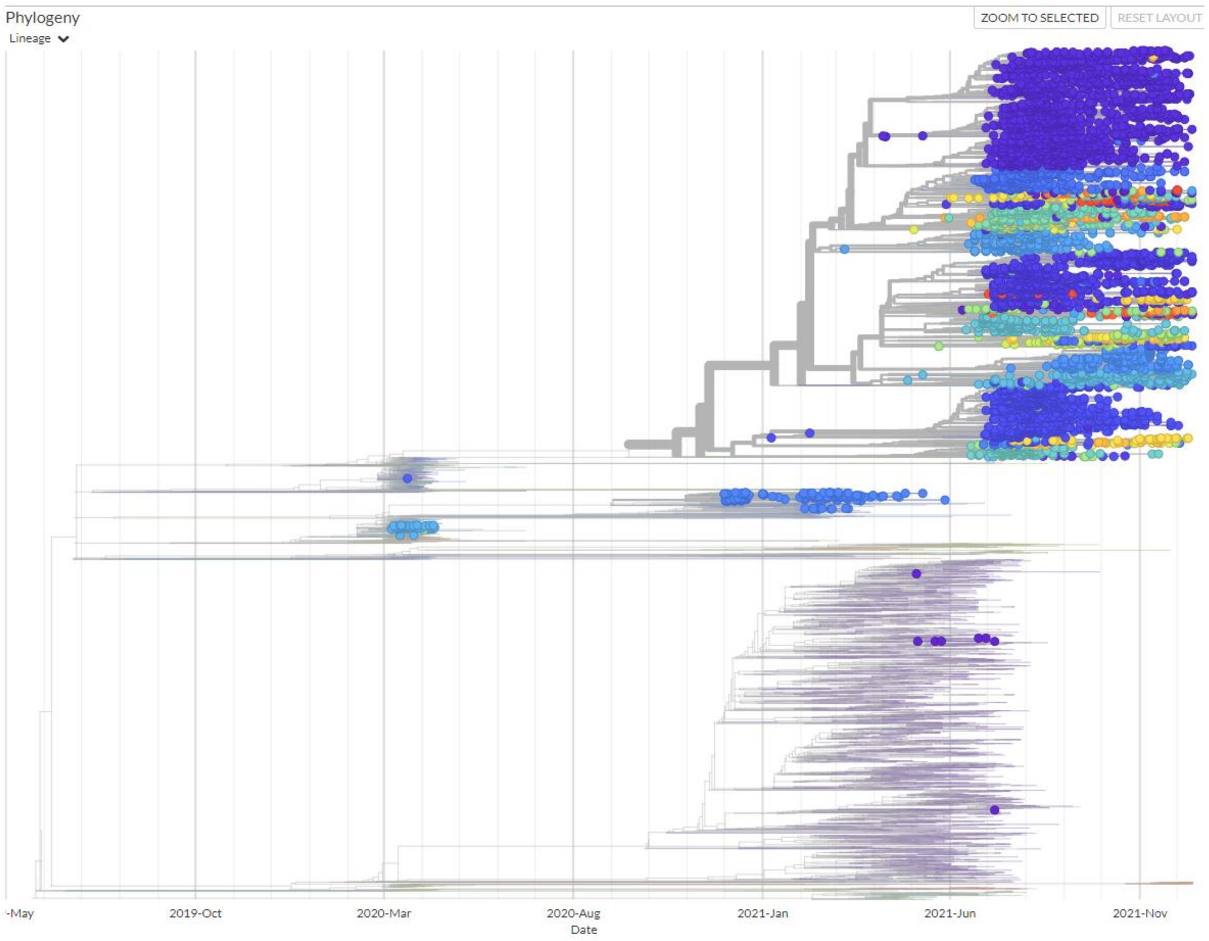

Supplementary Table S1.

ENA sample and project Ids of the SARS-CoV sequences used in this work

ENA sample ID	ENA project ID
SAMEA10306558	PRJEB44396
SAMEA10305326	PRJEB44396
SAMEA10305325	PRJEB44396
SAMEA10305315	PRJEB44396
SAMEA10305310	PRJEB44396
SAMEA10305307	PRJEB44396
SAMEA10305297	PRJEB44396
SAMEA10305293	PRJEB44396
SAMEA10304167	PRJEB44396
SAMEA10304151	PRJEB44396
SAMEA10304142	PRJEB44396
SAMEA10304135	PRJEB44396
SAMEA10304117	PRJEB44396
SAMEA10304111	PRJEB44396
SAMEA10304110	PRJEB44396
SAMEA10304108	PRJEB44396
SAMEA10304104	PRJEB44396
SAMEA10304101	PRJEB44396
SAMEA10304096	PRJEB44396
SAMEA10304090	PRJEB44396
SAMEA10304085	PRJEB44396
SAMEA10304083	PRJEB44396
SAMEA10304079	PRJEB44396
SAMEA10304078	PRJEB44396
SAMEA10304077	PRJEB44396
SAMEA10304076	PRJEB44396
SAMEA10304075	PRJEB44396
SAMEA10304074	PRJEB44396
SAMEA10304069	PRJEB44396
SAMEA10304068	PRJEB44396
SAMEA10304058	PRJEB44396
SAMEA10304056	PRJEB44396
SAMEA10304054	PRJEB44396
SAMEA10304049	PRJEB44396
SAMEA10304045	PRJEB44396
SAMEA10304043	PRJEB44396
SAMEA10304042	PRJEB44396
SAMEA10299902	PRJEB44396
SAMEA10299886	PRJEB44396
SAMEA10299795	PRJEB44396
SAMEA10299749	PRJEB44396
SAMEA10299528	PRJEB44396
SAMEA10299520	PRJEB44396

SAMEA10299515	PRJEB44396
SAMEA10299508	PRJEB44396
SAMEA10299504	PRJEB44396
SAMEA10299503	PRJEB44396
SAMEA10299478	PRJEB44396
SAMEA8729476	PRJEB44396
SAMEA8722672	PRJEB44396
SAMEA8722663	PRJEB44396
SAMEA8693734	PRJEB44396
SAMEA8693691	PRJEB44396
SAMEA8693687	PRJEB44396
SAMEA8693686	PRJEB44396
SAMEA8693373	PRJEB44396
SAMEA8693372	PRJEB44396
SAMEA8693366	PRJEB44396
SAMEA8693364	PRJEB44396
SAMEA8693359	PRJEB44396
SAMEA8693341	PRJEB44396
SAMEA8693324	PRJEB44396
SAMEA8693321	PRJEB44396
SAMEA8693310	PRJEB44396
SAMEA8693307	PRJEB44396
SAMEA8693301	PRJEB44396
SAMEA8693281	PRJEB44396
SAMEA8693279	PRJEB44396
SAMEA8693273	PRJEB44396
SAMEA8693269	PRJEB44396
SAMEA8693263	PRJEB44396
SAMEA8693261	PRJEB44396
SAMEA8693255	PRJEB44396
SAMEA8694675	PRJEB44396
SAMEA8694671	PRJEB44396
SAMEA8694660	PRJEB44396
SAMEA8694650	PRJEB44396
SAMEA8694642	PRJEB44396
SAMEA8694632	PRJEB44396
SAMEA8694629	PRJEB44396
SAMEA8694624	PRJEB44396
SAMEA8694617	PRJEB44396
SAMEA8694613	PRJEB44396
SAMEA8694602	PRJEB44396
SAMEA8694600	PRJEB44396
SAMEA8694592	PRJEB44396
SAMEA8694589	PRJEB44396
SAMEA8694587	PRJEB44396
SAMEA8694575	PRJEB44396
SAMEA8694571	PRJEB44396
SAMEA8694567	PRJEB44396

SAMEA8694566	PRJEB44396
SAMEA8694554	PRJEB44396
SAMEA8694547	PRJEB44396
SAMEA8694542	PRJEB44396
SAMEA8694540	PRJEB44396
SAMEA8694534	PRJEB44396
SAMEA8694530	PRJEB44396
SAMEA8694524	PRJEB44396
SAMEA8694523	PRJEB44396
SAMEA8694517	PRJEB44396
SAMEA8697543	PRJEB44396
SAMEA8697541	PRJEB44396
SAMEA8697539	PRJEB44396
SAMEA8697536	PRJEB44396
SAMEA8697528	PRJEB44396
SAMEA8697524	PRJEB44396
SAMEA8697515	PRJEB44396
SAMEA8697514	PRJEB44396
SAMEA8697512	PRJEB44396
SAMEA8697501	PRJEB44396
SAMEA8697496	PRJEB44396
SAMEA8697485	PRJEB44396
SAMEA8697478	PRJEB44396
SAMEA8697475	PRJEB44396
SAMEA8697471	PRJEB44396
SAMEA8697470	PRJEB44396
SAMEA8697469	PRJEB44396
SAMEA8697468	PRJEB44396
SAMEA8697465	PRJEB44396
SAMEA8697456	PRJEB44396
SAMEA8697436	PRJEB44396
SAMEA8697431	PRJEB44396
SAMEA8697430	PRJEB44396
SAMEA8697428	PRJEB44396
SAMEA8697421	PRJEB44396
SAMEA8697395	PRJEB44396
SAMEA8697393	PRJEB44396
SAMEA8697391	PRJEB44396
SAMEA8697381	PRJEB44396
SAMEA8697380	PRJEB44396
SAMEA8697379	PRJEB44396
SAMEA8697377	PRJEB44396
SAMEA8710066	PRJEB44396
SAMEA8710062	PRJEB44396
SAMEA8710061	PRJEB44396
SAMEA8710058	PRJEB44396
SAMEA8710052	PRJEB44396
SAMEA8710051	PRJEB44396

SAMEA8710042	PRJEB44396
SAMEA8710040	PRJEB44396
SAMEA8710035	PRJEB44396
SAMEA8710033	PRJEB44396
SAMEA8710030	PRJEB44396
SAMEA8710025	PRJEB44396
SAMEA8710011	PRJEB44396
SAMEA8709999	PRJEB44396
SAMEA8709995	PRJEB44396
SAMEA8709987	PRJEB44396
SAMEA8709965	PRJEB44396
SAMEA8709955	PRJEB44396
SAMEA8709947	PRJEB44396
SAMEA8709943	PRJEB44396
SAMEA8709938	PRJEB44396
SAMEA8709933	PRJEB44396
SAMEA8709930	PRJEB44396
SAMEA10299437	PRJEB44396
SAMEA10299436	PRJEB44396
SAMEA10299432	PRJEB44396
SAMEA10299426	PRJEB44396
SAMEA10299404	PRJEB44396
SAMEA10299402	PRJEB44396
SAMEA10299397	PRJEB44396
SAMEA10299386	PRJEB44396
SAMEA10299385	PRJEB44396
SAMEA8717253	PRJEB44396
SAMEA8717245	PRJEB44396
SAMEA8717244	PRJEB44396
SAMEA8717243	PRJEB44396
SAMEA8717237	PRJEB44396
SAMEA8717233	PRJEB44396
SAMEA8717226	PRJEB44396
SAMEA8717217	PRJEB44396
SAMEA8717211	PRJEB44396
SAMEA8717207	PRJEB44396
SAMEA8717189	PRJEB44396
SAMEA8717178	PRJEB44396
SAMEA8717164	PRJEB44396
SAMEA8717160	PRJEB44396
SAMEA8717157	PRJEB44396
SAMEA8717155	PRJEB44396
SAMEA8717151	PRJEB44396
SAMEA8717150	PRJEB44396
SAMEA8717136	PRJEB44396
SAMEA8717135	PRJEB44396
SAMEA8717129	PRJEB44396
SAMEA8717128	PRJEB44396

SAMEA8717123	PRJEB44396
SAMEA8712601	PRJEB44396
SAMEA8712588	PRJEB44396
SAMEA10299370	PRJEB44396
SAMEA10299369	PRJEB44396
SAMEA10299365	PRJEB44396
SAMEA10299357	PRJEB44396
SAMEA10299354	PRJEB44396
SAMEA10299349	PRJEB44396
SAMEA10299346	PRJEB44396
SAMEA10299339	PRJEB44396
SAMEA10299337	PRJEB44396
SAMEA10299334	PRJEB44396
SAMEA10299330	PRJEB44396
SAMEA10299326	PRJEB44396
SAMEA10299325	PRJEB44396
SAMEA10299320	PRJEB44396
SAMEA10299312	PRJEB44396
SAMEA10299307	PRJEB44396
SAMEA10299305	PRJEB44396
SAMEA10299303	PRJEB44396
SAMEA8724636	PRJEB44396
SAMEA8724634	PRJEB44396
SAMEA8724627	PRJEB44396
SAMEA8724625	PRJEB44396
SAMEA8724618	PRJEB44396
SAMEA8724602	PRJEB44396
SAMEA8724583	PRJEB44396
SAMEA8724572	PRJEB44396
SAMEA8724564	PRJEB44396
SAMEA8724563	PRJEB44396
SAMEA8724559	PRJEB44396
SAMEA8724555	PRJEB44396
SAMEA8724542	PRJEB44396
SAMEA8724536	PRJEB44396
SAMEA8724533	PRJEB44396
SAMEA8724526	PRJEB44396
SAMEA8724520	PRJEB44396
SAMEA8724519	PRJEB44396
SAMEA8724514	PRJEB44396
SAMEA8724513	PRJEB44396
SAMEA8724506	PRJEB44396
SAMEA10299300	PRJEB44396
SAMEA10299282	PRJEB44396
SAMEA10299259	PRJEB44396
SAMEA10299250	PRJEB44396
SAMEA10299244	PRJEB44396
SAMEA10299229	PRJEB44396

SAMEA10299223	PRJEB44396
SAMEA10291327	PRJEB44396
SAMEA10291238	PRJEB44396
SAMEA10291350	PRJEB44396
SAMEA10291333	PRJEB44396
SAMEA10291353	PRJEB44396
SAMEA10291326	PRJEB44396
SAMEA10291292	PRJEB44396
SAMEA10291323	PRJEB44396
SAMEA10291231	PRJEB44396
SAMEA10291343	PRJEB44396
SAMEA10291336	PRJEB44396
SAMEA10291281	PRJEB44396
SAMEA10291273	PRJEB44396
SAMEA10291227	PRJEB44396
SAMEA10291372	PRJEB44396
SAMEA10291369	PRJEB44396
SAMEA10291311	PRJEB44396
SAMEA10291263	PRJEB44396
SAMEA10291223	PRJEB44396
SAMEA10291254	PRJEB44396
SAMEA10291341	PRJEB44396
SAMEA10291247	PRJEB44396
SAMEA10291306	PRJEB44396
SAMEA10291214	PRJEB44396
SAMEA10291244	PRJEB44396
SAMEA10291340	PRJEB44396
SAMEA10291211	PRJEB44396
SAMEA10291347	PRJEB44396
SAMEA10291144	PRJEB44396
SAMEA10291140	PRJEB44396
SAMEA10291190	PRJEB44396
SAMEA10291160	PRJEB44396
SAMEA10291134	PRJEB44396
SAMEA10291133	PRJEB44396
SAMEA10291195	PRJEB44396
SAMEA10291131	PRJEB44396
SAMEA10291130	PRJEB44396
SAMEA10291122	PRJEB44396
SAMEA10291166	PRJEB44396
SAMEA10291118	PRJEB44396
SAMEA10291074	PRJEB44396
SAMEA10291073	PRJEB44396
SAMEA10291199	PRJEB44396
SAMEA10291174	PRJEB44396
SAMEA10291153	PRJEB44396
SAMEA10291102	PRJEB44396
SAMEA10291198	PRJEB44396

SAMEA10291094	PRJEB44396
SAMEA10291170	PRJEB44396
SAMEA10291088	PRJEB44396
SAMEA10291062	PRJEB44396
SAMEA10291081	PRJEB44396
SAMEA10291080	PRJEB44396
SAMEA10290991	PRJEB44396
SAMEA10290988	PRJEB44396
SAMEA10290984	PRJEB44396
SAMEA10291013	PRJEB44396
SAMEA10291010	PRJEB44396
SAMEA10291009	PRJEB44396
SAMEA10290976	PRJEB44396
SAMEA10290924	PRJEB44396
SAMEA10290975	PRJEB44396
SAMEA10291043	PRJEB44396
SAMEA10291007	PRJEB44396
SAMEA10290973	PRJEB44396
SAMEA10290896	PRJEB44396
SAMEA10290922	PRJEB44396
SAMEA10290970	PRJEB44396
SAMEA10291005	PRJEB44396
SAMEA10291047	PRJEB44396
SAMEA10291032	PRJEB44396
SAMEA10291042	PRJEB44396
SAMEA10290963	PRJEB44396
SAMEA10290960	PRJEB44396
SAMEA10290914	PRJEB44396
SAMEA10290955	PRJEB44396
SAMEA10290951	PRJEB44396
SAMEA10290998	PRJEB44396
SAMEA10290908	PRJEB44396
SAMEA10290948	PRJEB44396
SAMEA10290947	PRJEB44396
SAMEA10290995	PRJEB44396
SAMEA10291039	PRJEB44396
SAMEA10291026	PRJEB44396
SAMEA10291041	PRJEB44396
SAMEA10290994	PRJEB44396
SAMEA10291015	PRJEB44396
SAMEA10290993	PRJEB44396
SAMEA10290901	PRJEB44396
SAMEA10290992	PRJEB44396
SAMEA10290936	PRJEB44396
SAMEA10290898	PRJEB44396
SAMEA10290839	PRJEB44396
SAMEA10290872	PRJEB44396
SAMEA10290884	PRJEB44396

SAMEA10290870	PRJEB44396
SAMEA10290869	PRJEB44396
SAMEA10290879	PRJEB44396
SAMEA10290866	PRJEB44396
SAMEA10290864	PRJEB44396
SAMEA10290862	PRJEB44396
SAMEA10290877	PRJEB44396
SAMEA10290861	PRJEB44396
SAMEA10290860	PRJEB44396
SAMEA10290859	PRJEB44396
SAMEA10290858	PRJEB44396
SAMEA10290856	PRJEB44396
SAMEA10290831	PRJEB44396
SAMEA10290849	PRJEB44396
SAMEA10290848	PRJEB44396
SAMEA10290847	PRJEB44396
SAMEA10290887	PRJEB44396
SAMEA10290827	PRJEB44396
SAMEA10290888	PRJEB44396
SAMEA10290797	PRJEB44396
SAMEA10290803	PRJEB44396
SAMEA10290802	PRJEB44396
SAMEA10290826	PRJEB44396
SAMEA10290796	PRJEB44396
SAMEA10290825	PRJEB44396
SAMEA10290822	PRJEB44396
SAMEA10290817	PRJEB44396
SAMEA10290810	PRJEB44396
SAMEA10290807	PRJEB44396
SAMEA10290782	PRJEB44396
SAMEA10290544	PRJEB44396
SAMEA10290593	PRJEB44396
SAMEA10290610	PRJEB44396
SAMEA10290536	PRJEB44396
SAMEA10290535	PRJEB44396
SAMEA10290588	PRJEB44396
SAMEA10290532	PRJEB44396
SAMEA10290531	PRJEB44396
SAMEA10290583	PRJEB44396
SAMEA10290570	PRJEB44396
SAMEA10290568	PRJEB44396
SAMEA10290565	PRJEB44396
SAMEA10290501	PRJEB44396
SAMEA10253765	PRJEB47798
SAMEA10271074	PRJEB47798
SAMEA10271057	PRJEB47798
SAMEA10253708	PRJEB47798
SAMEA12009183	PRJEB47798

SAMEA10253774	PRJEB47798
SAMEA12009184	PRJEB47798
SAMEA10271085	PRJEB47798
SAMEA10253724	PRJEB47798
SAMEA12009185	PRJEB47798
SAMEA10253730	PRJEB47798
SAMEA10253788	PRJEB47798
SAMEA12009186	PRJEB47798
SAMEA12009187	PRJEB47798
SAMEA10253812	PRJEB47798
SAMEA10253764	PRJEB47798
SAMEA12009188	PRJEB47798
SAMEA10253750	PRJEB47798
SAMEA12009189	PRJEB47798
SAMEA10253715	PRJEB47798
SAMEA10253756	PRJEB47798
SAMEA10253864	PRJEB47798
SAMEA10253816	PRJEB47798
SAMEA10271075	PRJEB47798
SAMEA10253820	PRJEB47798
SAMEA10253768	PRJEB47798
SAMEA10253727	PRJEB47798
SAMEA10253736	PRJEB47798
SAMEA10253731	PRJEB47798
SAMEA10253748	PRJEB47798
SAMEA10253721	PRJEB47798
SAMEA10253728	PRJEB47798
SAMEA10253804	PRJEB47798
SAMEA10253819	PRJEB47798
SAMEA10253808	PRJEB47798
SAMEA10260231	PRJEB47798
SAMEA10260310	PRJEB47798
SAMEA10260295	PRJEB47798
SAMEA10260307	PRJEB47798
SAMEA10260239	PRJEB47798
SAMEA12009190	PRJEB47798
SAMEA10260316	PRJEB47798
SAMEA10260303	PRJEB47798
SAMEA12009191	PRJEB47798
SAMEA12009192	PRJEB47798
SAMEA12009193	PRJEB47798
SAMEA12009194	PRJEB47798
SAMEA12009195	PRJEB47798
SAMEA10271527	PRJEB47798
SAMEA10271530	PRJEB47798
SAMEA12009196	PRJEB47798
SAMEA10271511	PRJEB47798
SAMEA10271509	PRJEB47798

SAMEA10271526	PRJEB47798
SAMEA10271538	PRJEB47798
SAMEA10271507	PRJEB47798
SAMEA10271040	PRJEB47798
SAMEA12009197	PRJEB47798
SAMEA12009198	PRJEB47798
SAMEA10271043	PRJEB47798
SAMEA12009199	PRJEB47798
SAMEA10271550	PRJEB47798
SAMEA12009200	PRJEB47798
SAMEA10271553	PRJEB47798
SAMEA10271551	PRJEB47798
SAMEA10271049	PRJEB47798
SAMEA12009201	PRJEB47798
SAMEA10271052	PRJEB47798
SAMEA12009202	PRJEB47798
SAMEA10253840	PRJEB47798
SAMEA10253760	PRJEB47798
SAMEA10253725	PRJEB47798
SAMEA10253770	PRJEB47798
SAMEA10253755	PRJEB47798
SAMEA10253803	PRJEB47798
SAMEA10253779	PRJEB47798
SAMEA10253854	PRJEB47798
SAMEA10253801	PRJEB47798
SAMEA10253792	PRJEB47798
SAMEA10253836	PRJEB47798
SAMEA10253746	PRJEB47798
SAMEA10253862	PRJEB47798
SAMEA12009203	PRJEB47798
SAMEA10253845	PRJEB47798
SAMEA10253729	PRJEB47798
SAMEA10253815	PRJEB47798
SAMEA10253807	PRJEB47798
SAMEA12009204	PRJEB47798
SAMEA10253811	PRJEB47798
SAMEA10253800	PRJEB47798
SAMEA12009205	PRJEB47798
SAMEA10253777	PRJEB47798
SAMEA12009206	PRJEB47798
SAMEA10253844	PRJEB47798
SAMEA10253796	PRJEB47798
SAMEA10253810	PRJEB47798
SAMEA10253734	PRJEB47798
SAMEA10253817	PRJEB47798
SAMEA10253744	PRJEB47798
SAMEA10253758	PRJEB47798
SAMEA10253821	PRJEB47798

SAMEA10253710	PRJEB47798
SAMEA10253814	PRJEB47798
SAMEA10253754	PRJEB47798
SAMEA10253795	PRJEB47798
SAMEA12009207	PRJEB47798
SAMEA10253752	PRJEB47798
SAMEA10253809	PRJEB47798
SAMEA10270430	PRJEB47798
SAMEA10270449	PRJEB47798
SAMEA12009208	PRJEB47798
SAMEA10270541	PRJEB47798
SAMEA10270427	PRJEB47798
SAMEA10270412	PRJEB47798
SAMEA10270578	PRJEB47798
SAMEA10270556	PRJEB47798
SAMEA10270411	PRJEB47798
SAMEA10270505	PRJEB47798
SAMEA10270446	PRJEB47798
SAMEA10270555	PRJEB47798
SAMEA10270464	PRJEB47798
SAMEA10253719	PRJEB47798
SAMEA10270577	PRJEB47798
SAMEA10270445	PRJEB47798
SAMEA10270501	PRJEB47798
SAMEA12009209	PRJEB47798
SAMEA10270538	PRJEB47798
SAMEA10253848	PRJEB47798
SAMEA10253784	PRJEB47798
SAMEA10253847	PRJEB47798
SAMEA10253711	PRJEB47798
SAMEA10253789	PRJEB47798
SAMEA10253753	PRJEB47798
SAMEA10253794	PRJEB47798
SAMEA10253853	PRJEB47798
SAMEA10271175	PRJEB47798
SAMEA10271157	PRJEB47798
SAMEA10270500	PRJEB47798
SAMEA10270483	PRJEB47798
SAMEA10270484	PRJEB47798
SAMEA10270481	PRJEB47798
SAMEA10270480	PRJEB47798
SAMEA10270423	PRJEB47798
SAMEA12009210	PRJEB47798
SAMEA10270442	PRJEB47798
SAMEA12009211	PRJEB47798
SAMEA10270576	PRJEB47798
SAMEA10270441	PRJEB47798
SAMEA10270479	PRJEB47798

SAMEA12009212	PRJEB47798
SAMEA12009213	PRJEB47798
SAMEA10270574	PRJEB47798
SAMEA12009214	PRJEB47798
SAMEA10270572	PRJEB47798
SAMEA10270552	PRJEB47798
SAMEA10270458	PRJEB47798
SAMEA10270536	PRJEB47798
SAMEA10270438	PRJEB47798
SAMEA10270476	PRJEB47798
SAMEA10270518	PRJEB47798
SAMEA10270421	PRJEB47798
SAMEA10270517	PRJEB47798
SAMEA10271164	PRJEB47798
SAMEA10271145	PRJEB47798
SAMEA10271163	PRJEB47798
SAMEA10271078	PRJEB47798
SAMEA10271155	PRJEB47798
SAMEA12009215	PRJEB47798
SAMEA10271133	PRJEB47798
SAMEA10271103	PRJEB47798
SAMEA12009216	PRJEB47798
SAMEA10271165	PRJEB47798
SAMEA10271142	PRJEB47798
SAMEA10271129	PRJEB47798
SAMEA12009217	PRJEB47798
SAMEA10271153	PRJEB47798
SAMEA10271132	PRJEB47798
SAMEA10260299	PRJEB47798
SAMEA10260258	PRJEB47798
SAMEA12009218	PRJEB47798
SAMEA10260284	PRJEB47798
SAMEA10260264	PRJEB47798
SAMEA10260263	PRJEB47798
SAMEA10260267	PRJEB47798
SAMEA10271107	PRJEB47798
SAMEA10271160	PRJEB47798
SAMEA12009219	PRJEB47798
SAMEA10271121	PRJEB47798
SAMEA10271138	PRJEB47798
SAMEA10271545	PRJEB47798
SAMEA10271036	PRJEB47798
SAMEA12009220	PRJEB47798
SAMEA12009221	PRJEB47798
SAMEA10271547	PRJEB47798
SAMEA12009222	PRJEB47798
SAMEA10271544	PRJEB47798
SAMEA10260280	PRJEB47798

SAMEA10260247	PRJEB47798
SAMEA12009223	PRJEB47798
SAMEA10260240	PRJEB47798
SAMEA10260312	PRJEB47798
SAMEA10260287	PRJEB47798
SAMEA10260259	PRJEB47798
SAMEA10260245	PRJEB47798
SAMEA10260285	PRJEB47798
SAMEA10260260	PRJEB47798
SAMEA10260304	PRJEB47798
SAMEA12009224	PRJEB47798
SAMEA10260302	PRJEB47798
SAMEA10260308	PRJEB47798
SAMEA10260265	PRJEB47798
SAMEA12009225	PRJEB47798
SAMEA12009226	PRJEB47798
SAMEA10270453	PRJEB47798
SAMEA10270475	PRJEB47798
SAMEA12009227	PRJEB47798
SAMEA10270437	PRJEB47798
SAMEA10271180	PRJEB47798
SAMEA10253782	PRJEB47798
SAMEA10253793	PRJEB47798
SAMEA10253716	PRJEB47798
SAMEA10253757	PRJEB47798
SAMEA10271178	PRJEB47798
SAMEA10271120	PRJEB47798
SAMEA10271170	PRJEB47798
SAMEA10271156	PRJEB47798
SAMEA10271122	PRJEB47798
SAMEA10271126	PRJEB47798
SAMEA10271119	PRJEB47798
SAMEA10271151	PRJEB47798
SAMEA10271117	PRJEB47798
SAMEA10271137	PRJEB47798
SAMEA10271060	PRJEB47798
SAMEA10271110	PRJEB47798
SAMEA10271169	PRJEB47798
SAMEA10253850	PRJEB47798
SAMEA10271177	PRJEB47798
SAMEA10253837	PRJEB47798
SAMEA10271099	PRJEB47798
SAMEA10271091	PRJEB47798
SAMEA10271070	PRJEB47798
SAMEA12009228	PRJEB47798
SAMEA10270569	PRJEB47798
SAMEA10253858	PRJEB47798
SAMEA10270531	PRJEB47798

SAMEA10270493	PRJEB47798
SAMEA10260296	PRJEB47798
SAMEA10260237	PRJEB47798
SAMEA10260244	PRJEB47798
SAMEA12009229	PRJEB47798
SAMEA10260274	PRJEB47798
SAMEA10271130	PRJEB47798
SAMEA10271055	PRJEB47798
SAMEA10271058	PRJEB47798
SAMEA10271109	PRJEB47798
SAMEA10271125	PRJEB47798
SAMEA10271124	PRJEB47798
SAMEA10271108	PRJEB47798
SAMEA10271059	PRJEB47798
SAMEA10271105	PRJEB47798
SAMEA10271081	PRJEB47798
SAMEA10271063	PRJEB47798
SAMEA10271073	PRJEB47798
SAMEA10271100	PRJEB47798
SAMEA10271094	PRJEB47798
SAMEA12009230	PRJEB47798
SAMEA10271123	PRJEB47798
SAMEA10271090	PRJEB47798
SAMEA12009231	PRJEB47798
SAMEA10271096	PRJEB47798
SAMEA10271072	PRJEB47798
SAMEA10271089	PRJEB47798
SAMEA10271106	PRJEB47798
SAMEA10271064	PRJEB47798
SAMEA12009232	PRJEB47798
SAMEA10271093	PRJEB47798
SAMEA10270407	PRJEB47798
SAMEA10270416	PRJEB47798
SAMEA10270546	PRJEB47798
SAMEA10270514	PRJEB47798
SAMEA10270406	PRJEB47798
SAMEA10270452	PRJEB47798
SAMEA10270405	PRJEB47798
SAMEA10270513	PRJEB47798
SAMEA10270565	PRJEB47798
SAMEA10270512	PRJEB47798
SAMEA10270473	PRJEB47798
SAMEA8922088	PRJEB43166
SAMEA8922114	PRJEB43166
SAMEA8922109	PRJEB43166
SAMEA8922102	PRJEB43166
SAMEA8922091	PRJEB43166
SAMEA8922056	PRJEB43166

SAMEA8922046	PRJEB43166
SAMEA8922104	PRJEB43166
SAMEA8922092	PRJEB43166
SAMEA8922083	PRJEB43166
SAMEA8922078	PRJEB43166
SAMEA8922076	PRJEB43166
SAMEA8922037	PRJEB43166
SAMEA8922036	PRJEB43166
SAMEA8922094	PRJEB43166
SAMEA8922075	PRJEB43166
SAMEA8922098	PRJEB43166
SAMEA8922117	PRJEB43166
SAMEA8922113	PRJEB43166
SAMEA8922112	PRJEB43166
SAMEA8922110	PRJEB43166
SAMEA8922108	PRJEB43166
SAMEA8922097	PRJEB43166
SAMEA8922096	PRJEB43166
SAMEA8922095	PRJEB43166
SAMEA8922093	PRJEB43166
SAMEA8922089	PRJEB43166
SAMEA8922087	PRJEB43166
SAMEA8922086	PRJEB43166
SAMEA8922085	PRJEB43166
SAMEA8922084	PRJEB43166
SAMEA8922082	PRJEB43166
SAMEA8922080	PRJEB43166
SAMEA8922079	PRJEB43166
SAMEA8922074	PRJEB43166
SAMEA8922073	PRJEB43166
SAMEA8922041	PRJEB43166
SAMEA8922090	PRJEB43166
SAMEA8922081	PRJEB43166
SAMEA8921680	PRJEB43166
SAMEA8921678	PRJEB43166
SAMEA8921676	PRJEB43166
SAMEA8921284	PRJEB43166
SAMEA8921282	PRJEB43166
SAMEA8921280	PRJEB43166
SAMEA8921277	PRJEB43166
SAMEA8921271	PRJEB43166
SAMEA8921266	PRJEB43166
SAMEA8921263	PRJEB43166
SAMEA8921260	PRJEB43166
SAMEA8921254	PRJEB43166
SAMEA8921247	PRJEB43166
SAMEA8921245	PRJEB43166
SAMEA8921232	PRJEB43166

SAMEA8921229	PRJEB43166
SAMEA8921228	PRJEB43166
SAMEA8921221	PRJEB43166
SAMEA8921216	PRJEB43166
SAMEA8921215	PRJEB43166
SAMEA8921214	PRJEB43166
SAMEA8921211	PRJEB43166
SAMEA8921206	PRJEB43166
SAMEA8921204	PRJEB43166
SAMEA8921608	PRJEB43166
SAMEA8921378	PRJEB43166
SAMEA8921366	PRJEB43166
SAMEA8921203	PRJEB43166
SAMEA8921201	PRJEB43166
SAMEA8921200	PRJEB43166
SAMEA8921199	PRJEB43166
SAMEA8921198	PRJEB43166
SAMEA8921197	PRJEB43166
SAMEA8921192	PRJEB43166
SAMEA8921187	PRJEB43166
SAMEA8921186	PRJEB43166
SAMEA8921185	PRJEB43166
SAMEA8921181	PRJEB43166
SAMEA8921179	PRJEB43166
SAMEA8921165	PRJEB43166
SAMEA8921163	PRJEB43166
SAMEA8921162	PRJEB43166
SAMEA8921160	PRJEB43166
SAMEA8921158	PRJEB43166
SAMEA8921156	PRJEB43166
SAMEA8921155	PRJEB43166
SAMEA8921154	PRJEB43166
SAMEA8921153	PRJEB43166
SAMEA8921152	PRJEB43166
SAMEA8921151	PRJEB43166
SAMEA8921150	PRJEB43166
SAMEA8921138	PRJEB43166
SAMEA8921135	PRJEB43166
SAMEA8921132	PRJEB43166
SAMEA8921130	PRJEB43166
SAMEA8922043	PRJEB43166
SAMEA8922115	PRJEB43166
SAMEA8922107	PRJEB43166
SAMEA8922067	PRJEB43166
SAMEA8922100	PRJEB43166
SAMEA8922099	PRJEB43166
SAMEA8922105	PRJEB43166
SAMEA8922070	PRJEB43166

SAMEA8921372	PRJEB43166

Supplementary Table S2.

Nucleotide mutations eligible for causal analysis. The first column is the mutation name; the second is the position; the third column, labeled as CDS, is the protein affected; the fourth column is the amino acid mutation name; the fifth column is the number of variants bearing this mutation; and the following columns provide the values of the two approaches for hazard ratio estimation, the closed form, with the hazard ratio coefficient, SD, confidence intervals 5 and 95, the p-value and the FDR adjusted p-value, and the bootstrap approach with the HR coefficients (Boot. Statistic), bias, SD, confidence intervals 5 and 95 and the last column, labeled as Boot, indicates if significance is confirmed by bootstrap (T: true and F: false)

Mutation name	position	CDS	AAc mutation name	cases	Theor coeff.	Theor. StD	Theor. CI05	Theor. CI95	Theor. P-val	Theor. P-val FDR	Boot. statistic	Boot bias	Boot. Std Err	Boot CI 05	Boot CI 95	Boot
28274	28274	N	N:M1M	291	0.5614	0.1839	0.2010	0.9219	0.0023	0.0423	0.5614	0.0030	0.1940	0.1832	0.9322	T
C5388A	5388	ORF1ab	ORF1ab:A1708D	288	0.5213	0.1835	0.1616	0.8811	0.0045	0.0423	0.5213	-0.0061	0.1873	0.1795	0.9136	T
C23709T	23709	S	S:T716I	294	0.5179	0.1827	0.1598	0.8761	0.0046	0.0423	0.5179	-0.0058	0.1912	0.1450	0.8933	T
T6954C	6954	ORF1ab	ORF1ab:I2230T	284	0.5208	0.1843	0.1595	0.8821	0.0047	0.0423	0.5208	0.0037	0.1892	0.1345	0.8931	T
C14676T	14676	ORF1ab	ORF1ab:P4803P	288	0.5136	0.1834	0.1542	0.8731	0.0051	0.0423	0.5136	0.0019	0.1867	0.0840	0.8374	T
C15279T	15279	ORF1ab	ORF1ab:H5004H	288	0.5049	0.1824	0.1474	0.8625	0.0056	0.0423	0.5049	-0.0107	0.1932	0.1358	0.8936	T
C23604A	23604	S	S:P681H	296	0.5033	0.1828	0.1450	0.8615	0.0059	0.0423	0.5033	-0.0062	0.1902	0.1215	0.8759	T
11288,11297	11288	ORF1ab	ORF1ab:del3675-3677	291	0.4957	0.1831	0.1368	0.8545	0.0068	0.0423	0.4957	0.0022	0.1906	0.1271	0.8695	T
C23271A	23271	S	S:A570D	289	0.4853	0.1821	0.1283	0.8422	0.0077	0.0423	0.4853	-0.0093	0.1863	0.1368	0.8545	T
G28280C	28280	N	N:D3L	288	0.4856	0.1838	0.1254	0.8458	0.0082	0.0423	0.4856	-0.0021	0.1858	0.1307	0.8725	T
A28281T	28281	N	N:D3L	288	0.4856	0.1838	0.1254	0.8458	0.0082	0.0423	0.4856	-0.0021	0.1858	0.1307	0.8725	T
T28282A	28282	N	N:D3L	288	0.4856	0.1838	0.1254	0.8458	0.0082	0.0423	0.4856	-0.0021	0.1858	0.1307	0.8725	T
A28111G	28111	ORF8	ORF8:Y73C	288	0.4863	0.1846	0.1244	0.8481	0.0084	0.0423	0.4863	-0.0050	0.1923	0.1458	0.9159	T
C913T	913	ORF1ab	ORF1ab:S216S	288	0.4805	0.1828	0.1221	0.8388	0.0086	0.0423	0.4805	-0.0078	0.1878	0.1423	0.8535	T
T24506G	24506	S	S:S982A	290	0.4721	0.1829	0.1136	0.8306	0.0099	0.0442	0.4721	-0.0002	0.1874	0.0670	0.8295	T
C5986T	5986	ORF1ab	ORF1ab:F1907F	288	0.4677	0.1822	0.1106	0.8247	0.0102	0.0442	0.4677	-0.0043	0.1892	0.0976	0.8338	T
C3267T	3267	ORF1ab	ORF1ab:T1001I	290	0.4612	0.1818	0.1049	0.8175	0.0112	0.0454	0.4612	0.0015	0.1903	0.0775	0.8307	T
G24914C	24914	S	S:D1118H	288	0.4604	0.1832	0.1013	0.8194	0.0120	0.0459	0.4604	0.0073	0.1904	0.0846	0.8228	T
21766,21772	21766	S	S:del69-70	280	0.4567	0.1836	0.0969	0.8164	0.0128	0.0467	0.4567	0.0027	0.1942	0.0495	0.8196	T
G28048T	28048	ORF8	ORF8:R52I	289	0.4571	0.1852	0.0940	0.8201	0.0136	0.0468	0.4571	-0.0038	0.1857	0.1008	0.8255	T
C27972T	27972	ORF8	ORF8:Q27*	290	0.4515	0.1853	0.0883	0.8147	0.0148	0.0468	0.4515	0.0009	0.1875	0.0635	0.8169	T

C28977T	28977	N	N:S235F	290	0.4448	0.1827	0.0867	0.8029	0.0149	0.0468	0.4448	-0.0038	0.1889	0.0531	0.8014	T
T16176C	16176	ORF1ab	ORF1ab:T5303T	285	0.4390	0.1831	0.0801	0.7980	0.0165	0.0496	0.4390	-0.0070	0.1855	0.0609	0.8025	T
21994,21997	21994	S	S:Y144-	270	0.4431	0.1874	0.0758	0.8104	0.0181	0.0519	0.4431	0.0050	0.1944	0.0663	0.8194	T
A23063T	23063	S	S:N501Y	273	0.4380	0.1901	0.0654	0.8107	0.0212	0.0586	0.4380	-0.0080	0.1997	0.0314	0.8267	T
C10833T	10833	ORF1ab	ORF1ab:A3523V	26	-0.9421	0.5182	-1.9578	0.0736	0.0691	0.1833	-0.9421	-0.9488	4.1888	-21.3190	-0.0541	T
C6968T	6968	ORF1ab	ORF1ab:L2235L	71	0.4784	0.2707	-0.0521	1.0089	0.0772	0.1972	0.4784	-0.0019	0.2972	-0.1305	1.0217	F
G28882A	28882	NA	N:R203L	315	0.3026	0.1807	-0.0515	0.6567	0.0940	0.2235	0.3026	0.0027	0.1783	-0.0528	0.6544	F
G28883C	28883	N	N:G204R	315	0.3026	0.1807	-0.0515	0.6567	0.0940	0.2235	0.3026	0.0027	0.1783	-0.0528	0.6544	F
C22227T	22227	S	S:A222V	73	-0.4460	0.2846	-1.0039	0.1118	0.1171	0.2606	-0.4460	-0.0298	0.3076	-1.0691	0.1359	F
G28881A	28881	N	N:R203L	317	0.2829	0.1805	-0.0709	0.6366	0.1171	0.2606	0.2829	0.0027	0.1777	-0.0683	0.6288	F
G21255C	21255	ORF1ab	ORF1ab:A6996A	71	-0.4022	0.2614	-0.9145	0.1100	0.1238	0.2636	-0.4022	-0.0248	0.2838	-1.0252	0.1064	F
C222T	222	No ORF	No ORF	32	-0.7169	0.4687	-1.6355	0.2016	0.1261	0.2636	-0.7169	-0.3770	2.4235	-2.2067	0.1464	F
G4300T	4300	ORF1ab	ORF1ab:V1345V	5	1.3628	1.0428	-0.6811	3.4067	0.1913	0.3882	1.3628	-6.4484	12.3659	-21.2936	21.1406	F
C6286T	6286	ORF1ab	ORF1ab:T2007T	74	-0.3261	0.2598	-0.8353	0.1831	0.2095	0.4058	-0.3261	-0.0211	0.2668	-0.8521	0.1708	F
G1820A	1820	ORF1ab	ORF1ab:G519S	14	0.4874	0.3944	-0.2856	1.2603	0.2165	0.4058	0.4874	-0.0498	1.0898	-0.5423	1.3784	F
A17615G	17615	ORF1ab	ORF1ab:K5783R	66	0.4193	0.3401	-0.2473	1.0860	0.2176	0.4058	0.4193	-0.0381	0.3852	-0.3624	1.1394	F
C2710T	2710	ORF1ab	ORF1ab:L815L	12	0.6595	0.6019	-0.5201	1.8392	0.2732	0.4948	0.6595	-2.9978	7.6816	-21.3015	2.1132	F
G29734C	29734	No ORF	No ORF	46	0.3360	0.3129	-0.2772	0.9493	0.2828	0.4948	0.3360	-0.0098	0.3497	-0.3691	0.9999	F
A23403G	23403	S	D614G	616	0.2413	0.2266	-0.2028	0.6854	0.2868	0.4948	0.2413	0.0166	0.2407	-0.1949	0.7073	F
T445C	445	ORF1ab	ORF1ab:V60V	77	-0.2506	0.2437	-0.7283	0.2271	0.3038	0.5113	-0.2506	-0.0131	0.2589	-0.7613	0.2766	F
C28932T	28932	N, ORF9c	N:A220V, ORF9c:L67F	71	-0.2501	0.2557	-0.7513	0.2511	0.3281	0.5119	-0.2501	-0.0230	0.2657	-0.7788	0.2541	F
C26801G	26801	M	M:L93L	72	-0.2469	0.2560	-0.7487	0.2549	0.3349	0.5119	-0.2469	-0.0206	0.2745	-0.8157	0.2896	F
C14408T	14408	ORF1ab	ORF1ab:P4713L	615	0.2193	0.2275	-0.2266	0.6653	0.3350	0.5119	0.2193	0.0260	0.2498	-0.2387	0.7457	F
T29589C	29589	ORF10	ORF10:F11S	75	0.2732	0.2837	-0.2830	0.8293	0.3357	0.5119	0.2732	-0.0163	0.3053	-0.4343	0.8070	F
G29645T	29645	ORF10	ORF10:V30L	71	-0.2428	0.2551	-0.7428	0.2572	0.3413	0.5119	-0.2428	-0.0287	0.2715	-0.7374	0.3042	F
C25463A	25463	ORF3a, ORF3c	ORF3a:T24N, ORF3c:L3I	12	0.5123	0.6054	-0.6742	1.6989	0.3974	0.5779	0.5123	-2.5029	7.1754	-21.4067	2.4443	F
C25714A	25714	ORF3a	ORF3a:L108I	7	-0.3967	0.4734	-1.3246	0.5312	0.4021	0.5779	-0.3967	-2.5603	7.1736	-21.9799	1.1567	F
C16887T	13887	ORF1ab	ORF1ab:Y5540Y	79	0.2138	0.2701	-0.3156	0.7432	0.4286	0.5985	0.2138	-0.0139	0.2854	-0.3846	0.7693	F
G12892A	12892	ORF1ab	ORF1ab:L4209L	14	-0.7716	0.9856	-2.7034	1.1602	0.4337	0.5985	-0.7716	-7.0538	10.0145	-21.5310	0.9286	F
C8782T	8782	ORF1ab	ORF1ab:S2839S	142	-0.1813	0.2362	-0.6443	0.2817	0.4428	0.5991	-0.1813	-0.0228	0.2408	-0.6376	0.3152	F
T28144C	28144	ORF8	ORF8:L84S	145	-0.1690	0.2308	-0.6214	0.2835	0.4642	0.6159	-0.1690	-0.0129	0.2394	-0.6670	0.2782	F

C241T	241	No ORF	No ORF	624	0.1596	0.2238	-0.2789	0.5982	0.4756	0.6192	0.1596	0.0151	0.2354	-0.3052	0.6408	F
C3037T	3037	ORF1ab	ORF1ab:F924F	624	0.1554	0.2237	-0.2831	0.5939	0.4872	0.6225	0.1554	0.0186	0.2355	-0.2878	0.6425	F
C13671T	13671	ORF1ab	ORF1ab:F4468F	7	0.2569	0.4288	-0.5836	1.0974	0.5491	0.6889	0.2569	-0.9971	4.8332	-21.4026	1.7911	F
C27944T	27944	ORF8	ORF8:H17H	26	0.1639	0.3605	-0.5426	0.8705	0.6492	0.7999	0.1639	-0.0351	0.4125	-0.7527	0.8791	F
C28863T	28863	N, ORF9c	N:S197L, ORF9c:Q44-	122	-0.0885	0.2420	-0.5628	0.3857	0.7145	0.8649	-0.0885	-0.0135	0.2586	-0.6829	0.3750	F
C28657T	28657	N	N:D128D	120	-0.0692	0.2399	-0.5394	0.4010	0.7730	0.8987	-0.0692	-0.0270	0.2406	-0.5288	0.4204	F
C5833G	5833	ORF1ab	ORF1ab:S1856S	6	-0.1089	0.4240	-0.9399	0.7221	0.7973	0.8987	-0.1089	-2.3846	6.9040	-21.5059	1.8958	F
T6552C	6552	ORF1ab	ORF1ab:M2096T	12	0.2084	0.8795	-1.5154	1.9322	0.8127	0.8987	0.2084	-7.5357	10.3159	-21.1259	1.7815	F
G25979T	25979	ORF3a	ORF3a:G196V	121	-0.0537	0.2408	-0.5258	0.4183	0.8235	0.8987	-0.0537	-0.0180	0.2527	-0.5356	0.4367	F
21766,21771	21766	S	S:del69	7	0.1781	0.8144	-1.4181	1.7744	0.8269	0.8987	0.1781	-7.4589	10.5512	-21.3706	3.0984	F
A20268G	20268	ORF1ab	ORF1ab:L6667L	165	0.0417	0.2044	-0.3589	0.4422	0.8383	0.8987	0.0417	-0.0081	0.2132	-0.3802	0.4344	F
C14805T	14805	ORF1ab	ORF1ab:Y4846Y	121	-0.0440	0.2401	-0.5146	0.4266	0.8546	0.8987	-0.0440	-0.0082	0.2502	-0.6118	0.4066	F
G25647T	25647	ORF3a, ORF3d	ORF3a_L85F, ORF3d:V42F	12	0.1429	0.8582	-1.5391	1.8249	0.8678	0.8987	0.1429	-7.5487	10.3007	-21.1876	1.8360	F
G26262A	26262	M	M:S6S	12	0.1431	0.8606	-1.5436	1.8299	0.8679	0.8987	0.1431	-7.0384	10.1695	-21.2036	1.5148	F
C28725T	28725	N	N:P151L	12	0.1381	0.8618	-1.5509	1.8272	0.8726	0.8987	0.1381	-7.4038	10.2820	-21.1775	1.8618	F
G21974T	21974	S	S:D138Y	12	-0.0374	0.3523	-0.7280	0.6531	0.9154	0.9288	-0.0374	-1.4007	5.2867	-21.3816	0.8658	F
T9477A	9477	ORF1ab	ORF1ab:F3071Y	116	-0.0138	0.2397	-0.4836	0.4560	0.9540	0.9540	-0.0138	-0.0046	0.2531	-0.5382	0.4574	F

The Andalusian COVID-19 sequencing initiative

Francisco J Morón, Rosana March-Diaz, Salud Borrego, Irene Marcos: Institute of Biomedicine of Seville (IBIS), Hospital Virgen del Rocio. 41013. Sevilla. Spain;

Monica Perez-Alegre, Eloisa Andújar: Centro Andaluz de Biología Molecular y Medicina Regenerativa CABIMER, Universidad de Sevilla-CSIC-Universidad Pablo de Olavide, Sevilla, Spain.

Matilde Palanca Gimenez: Hospital Poniente de Almería, El Ejido, Almería, Spain

Manuel Rodríguez Maresca: Hospital Torrecárdenas, Almería, Spain

Manuel A. Rodríguez Iglesias: Hospital Puerta del Mar, Cádiz, Spain

Manuel Causse del Río, Cristina Riazzo, Luis Martínez-Martínez: Hospital Universitario Reina Sofía, Córdoba, Spain

Francisco Franco Álvarez De Luna: Hospital Juan Ramón Jiménez, Huelva, Spain

Carolina Roldán Fontana: Complejo Hospitalario de Jaén, Jaén, Spain

María Dolores López Prieto: Hospital de Jerez, Cádiz, Spain

Maria Luisa Hortas, Fernando Fernandez Sanchez: Hospital Costa del Sol, Málaga, Spain

Begoña Palop Borrás: Hospital Regional, Málaga, Spain

Isabel Viciana: Hospital Virgen de la Victoria, Málaga, Spain

Alvaro Pascual: Hospital Virgen de la Macarena, Sevilla; Institute of Biomedicine of Seville (IBIS), Hospital Virgen del Rocio. 41013. Sevilla. Spain

Ángel Rodríguez Villodres: Hospital Universitario Virgen del Rocío, Sevilla, Spain

Samuel Bernal Martinez, Estrella Martin Mazuelos: Unidad Clinica de Enfermedades Infecciosas y Microbiología (UCEIM), H.U. Virgen de Valme, Sevilla

Inés Ruiz Molina: Hospital Punta Europa, Cádiz, Spain

Natalia Chueca, Ana Fuentes: Servicio de Microbiología. Unidad Clínica Enfermedades Infecciosas, Microbiología y Medicina Preventiva. Hospital Universitario Virgen del Rocio. Sevilla. Spain
